# Distinct neural systems underlying reduced emotional enhancement for positive and negative stimuli in early Alzheimer's disease

**DOI:** 10.3389/fnhum.2013.00939

**Published:** 2014-01-20

**Authors:** Panagiota Mistridis, Kirsten I. Taylor, Johanna M. Kissler, Andreas U. Monsch, Reto W. Kressig, Sasa L. Kivisaari

**Affiliations:** ^1^Memory Clinic, University Center for Medicine of Aging Basel, Felix Platter HospitalBasel, Switzerland; ^2^Department of Psychology, University of BaselBasel, Switzerland; ^3^University Center for Medicine of Aging Basel, Felix Platter HospitalBasel, Switzerland; ^4^Department of Experimental Psychology, Centre for Speech, Language and the Brain, University of CambridgeCambridge, UK; ^5^Department of Psychology, University of BielefeldBielefeld, Germany; ^6^Department of Psychology, University of KonstanzKonstanz, Germany

**Keywords:** emotional memory, lateralization, Alzheimer's disease, amygdala, prefrontal cortex, angular gyrus

## Abstract

Emotional information is typically better remembered than neutral content, and previous studies suggest that this effect is subserved particularly by the amygdala together with its interactions with the hippocampus. However, it is not known whether amygdala damage affects emotional memory performance at immediate and delayed recall, and whether its involvement is modulated by stimulus valence. Moreover, it is unclear to what extent more distributed neocortical regions involved in e.g., autobiographical memory, also contribute to emotional processing. We investigated these questions in a group of patients with Alzheimer's disease (AD), which affects the amygdala, hippocampus and neocortical regions. Healthy controls (*n* = 14), patients with AD (*n* = 15) and its putative prodrome amnestic mild cognitive impairment (*n* = 11) completed a memory task consisting of immediate and delayed free recall of a list of positive, negative and neutral words. Memory performance was related to brain integrity in region of interest and whole-brain voxel-based morphometry analyses. In the brain-behavioral analyses, the left amygdala volume predicted the immediate recall of both positive and negative material, whereas at delay, left and right amygdala volumes were associated with performance with positive and negative words, respectively. Whole-brain analyses revealed additional associations between left angular gyrus integrity and the immediate recall of positive words as well as between the orbitofrontal cortex and the delayed recall of negative words. These results indicate that emotional memory impairments in AD may be underpinned by damage to regions implicated in emotional processing as well as frontoparietal regions, which may exert their influence via autobiographical memories and organizational strategies.

## Introduction

Events colored with positive or negative associations are better remembered than those that do not carry any emotional significance (Cahill and McGaugh, 1998; Kensinger and Corkin, 2003; Phelps, 2006). This emotional enhancement effect (Kensinger, 2006) has been suggested to be primarily driven by amygdala-modulated consolidation processes in the medial temporal lobe (Kensinger et al., 2002; Kensinger and Corkin, 2004; Ritchey et al., 2008). However, it is not known if the amygdala is also necessary for the short-term, i.e., immediate recall of emotional information, and whether its involvement in emotional memory is modulated by stimulus valence. Moreover, it is unclear to what extent damage to more distributed brain regions, which have been implicated in emotional processing (Dolcos et al., 2004a; Kensinger and Corkin, 2004; Smith et al., 2004; Kumfor et al., 2013) and autobiographical memory (Fink et al., 1996; Piefke et al., 2003) such as frontal lobe regions including the orbitofrontal cortex, are associated with emotional memory impairments at immediate and delayed recall. In the present study, we therefore examined whether amygdala volume is significantly related to immediate and delayed episodic memory performance with positive and negative emotional stimuli. We additionally performed whole-brain voxel-based analyses to determine the entire set of brain regions associated with emotional memory performance with positive and negative valences. We studied these questions in the context of Alzheimer's disease (AD) dementia, which is associated with neurofibrillary pathology in the amygdala and neocortical regions (Braak and Braak, 1991), as well as emotional memory impairments (Kensinger, 2006).

The importance of the amygdala for emotional long-term memory is well-established (Cahill and McGaugh, 1998; McGaugh, 2000; Phelps, 2006; Ritchey et al., 2008), and this region, together with its interactions with the hippocampus (LeDoux, 1993; Dolcos et al., 2004b; Ritchey et al., 2008), is claimed to enhance consolidation and strengthen emotional memory traces over time (McGaugh, 2000; Phelps, 2004). The relevance of the amygdala for the consolidation of emotionally arousing material is supported by classical aversive conditioning studies (Davis, 1992; LeDoux, 1993), functional imaging studies in humans (Hamann et al., 1999; Dolcos et al., 2004b; Ritchey et al., 2008) and clinical data (Cahill et al., 1995; Adolphs et al., 1997), where patients with amygdala damage fail to profit from stimulus emotionality at delayed recall (but see also Phelps et al., 1997).

In addition to these time-dependent effects, the amygdala purportedly facilitates the on-line processing of emotional material by enhancing and directing perception and attention toward emotional stimuli (Anderson and Phelps, 2001; Phelps et al., 2006; De Martino et al., 2009; Jacobs et al., 2012). This attentional facilitation for emotionally salient stimuli putatively increases the probability of these stimuli being stored (LaBar and Cabeza, 2006) and may partly account for the beneficial effects of emotionality on memory performance. Thus, this claim leads to the prediction that amygdala damage impairs not only the delayed, but also the immediate recall of emotional material. This prediction has to our knowledge not been explicitly tested and it is not known if the possible involvement of the amygdala at immediate recall is modulated by stimulus valence.

Imaging studies provide evidence for the involvement of the amygdala in the emotional enhancement of both positive and negative stimuli (Hamann et al., 1999; Garavan et al., 2001; Hamann and Mao, 2002). According to the hemispheric lateralization hypothesis, the left and right hemispheres process positive and negative material, respectively (Sackeim et al., 1982; Silberman and Weingartner, 1986). However, few studies supporting the hemispheric lateralization hypothesis directly compared psycholinguistically matched sets of positive and negative stimuli, such that it remains unclear whether or not such a lateralization exists in the amygdala. Some functional imaging studies which directly compared positive and negative material found the predicted lateralized pattern of amygdala activation (Canli et al., 1998; Zalla et al., 2000), while others reported bilateral (Garavan et al., 2001; Yang et al., 2002) or left-lateralized amygdala activation for both valences (Schneider et al., 1997; Hamann and Mao, 2002). Consistent with this latter finding, the majority of studies examining emotional processing (Morris et al., 1998; Canli et al., 2000; Zalla et al., 2000) and long-term memory (LaBar and Phelps, 1998; Adolphs et al., 2000; Buchanan et al., 2001; Frank and Tomaz, 2003) for either negative or positive material found primarily left-sided amygdala involvement for both valences (for an overview see Wager et al., 2003; Zald, 2003; Beraha et al., 2012). Thus, these results suggest that the right amygdala may play a weaker role in emotional memory enhancement compared to the left amygdala (LaBar and Phelps, 1998; Adolphs et al., 2000; Buchanan et al., 2001; Meletti et al., 2003, 2009) and that the processing of negative stimuli may be subserved by the bilateral amygdalae (Liberzon et al., 2000; Hamann et al., 2002; Ritchey et al., 2008; Baeken et al., 2009).

The emotional enhancement effect may also be driven by neuroanatomical systems outside medial temporal lobe structures, such as the prefrontal cortex including the orbitofrontal cortex (Kensinger and Corkin, 2004; Murty et al., 2010; Kumfor et al., 2013) and other neocortical regions (Fink et al., 1996; Piefke et al., 2003; Holland and Kensinger, 2010). Emotional items are thought to benefit from e.g., controlled (i.e., self-initiated and conscious) processes and encoding strategies such as rehearsal and elaborative semantic processing putatively supported by the prefrontal cortex (Kensinger, 2004; Kensinger and Corkin, 2004; Mather, 2007). Moreover, memory for emotional material may evoke recollections of personal past events by their close relationship to one's own autobiographical history. Thereby, emotional material may be more readily integrated with context-rich autobiographical memories than neutral material, since the former are particularly personally relevant to each individual (Holland and Kensinger, 2010). Autobiographical memory processing is claimed to engage a distributed network of brain regions including prefrontal and parietal regions and the cingulate cortex in addition to the medial temporal lobe (Fink et al., 1996; Piefke et al., 2003; Denkova et al., 2006; Steinvorth et al., 2006). Thus, regions implicated in self-controlled, organizational and autobiographical processes may be important for the emotional enhancement effect, and damage to these regions may indirectly impair emotional memory processing.

We examined these questions in the context of AD which is associated with neurofibrillary pathology in the medial temporal lobe, including the amygdala and hippocampus, and neocortical regions including the prefrontal cortex (Braak and Braak, 1991; Riley et al., 2002; Thompson et al., 2003) as well as with emotional memory impairments (Abrisqueta-Gomez et al., 2002; Kensinger et al., 2002, 2004; Borg et al., 2011). Specifically, we tested whether atrophy of the left and right amygdalae affects both immediate and delayed recall of emotional material, and whether valence modulates these relationships. Based on previous research, we predicted that performance with positive material is related to left amygdala volume, whereas negative material is related to left and right amygdala volumes, both at immediate and delayed recall. We directly tested these hypotheses using anatomical region of interest (ROI) analyses. To examine the entire set of brain regions associated with immediate and delayed recall of emotional material, we additionally related performance with positive and negative stimuli with the integrity of the whole brain and explored all regions that were uniquely associated with emotional processing of one valence (positive or negative) over and above that of the opposing valence and performance with neutral words. These results would contribute to a better understanding of the interactions between emotion and episodic memory and provide insights into the neuroanatomical correlates of emotional learning and memory processing in AD.

## Materials and methods

### Participants

Data from 40 German-speaking participants were included in this study [mean age = 73.05 years, standard deviation (SD) = 6.67 years; mean education = 11.75 years, *SD* = 2.81 years; 20 women, 20 men; mean Mini Mental Examination score (MMSE; Folstein et al., 1975) = 27.7, *SD* = 2.34]. Fourteen were neurologically and cognitively healthy individuals [normal control participants (NC)] selected from longitudinal studies on aging and dementia at the Memory Clinic Basel. All NC participants had undergone a comprehensive medical and neuropsychological examination to ensure their cognitive health. Eleven participants fulfilled the diagnostic criteria for amnestic mild cognitive impairment (aMCI) according to Winblad et al. (2004). Fifteen participants were diagnosed with AD according to the fourth edition of the Diagnostic and Statistical Manual of Mental Disorders (DSM-IV; American Psychiatric Association, 1994) and the National Institute of Neurological and Communicative Disorders and Stroke and the Alzheimer's Disease Related Disorders Association (NINCDS-ADRDA) criteria (McKhann et al., 1984). The three groups were comparable with respect to educational attainment and gender distribution, but differed significantly with respect to the MMSE score, as expected (see Table 1). Since group differences approached significance with respect to mean age, age was covaried in all statistical analyses (see Table 1). This study was approved by the ethics committee of both Basels and informed consent was obtained from each participant. A summary of participants' overall neuropsychological test performance is provided in the **Supplementary Methods**, Table S1.

**Table 1:** **Demographic characteristics and MMSE scores of the NC, aMCI, and AD groups [mean (standard deviation)]**.

	**Diagnosis**				
	**NC (*n* = 14)**	**aMCI (*n* = 11)**	**AD (*n* = 15)**	***F*/χ^2^**	***p*-value**
age, years	71.6 (6.1)	70.8 (6.3)	76.0 (6.9)	2.59	0.09
education, years	12.1 (2.2)	10.5 (2.4)	12.3 (3.4)	1.68	0.20
%women	35.7	54.5	60.0	1.83^a^	0.4
MMSE	29.3 (0.7)	27.8 (1.3)	25.9 (2.8)	11.54	<0.001

a χ^2^-test.

### Emotional memory task

#### Stimuli

Fifteen word stimuli were selected from a pool of 180 German nouns which had been rated by an independent sample of 45 student participants on the dimensions of valence and arousal (for details see Kissler et al., 2007). We selected a total of 15 words, as longer word lists would have overly taxed the memory impaired patients and as this length is comparable to other verbal memory tasks used in clinical neuropsychological assessment. One third of the words had a positive (*n* = 5, e.g., kiss), one third a negative (*n* = 5, e.g., pain), and one third a neutral valence (*n* = 5, e.g., pencil). The three valence categories differed significantly with respect to their mean valence ratings (positive > neutral > negative, all *p*-values < 0.001), while the mean arousal ratings of the positive and negative valence items were comparable [*t*_(8)_ = −1.84, *p* = 0.10] and differed significantly from neutral words [*t*_(8)_ = 8.10, *p* < 0.001; *t*_(8)_ = 12.63, *p* < 0.001, respectively]. The words were matched such that they did not differ with respect to concreteness, word length, number of phonemes or written word frequency (CELEX; Baayen et al., 1995). The means, standard deviations and comparisons of the psycholinguistic variables across emotional valence categories are presented in Table 2.

**Table 2:** **Emotional and psycholinguistic characteristics of the stimuli [mean (standard deviation)] and the results of an analysis of variance testing for differences between valence group means**.

	**Positive (*n* = 5)**	**Negative (*n* = 5)**	**Neutral (*n* = 5)**	***F***	***p*-value**
Valence^a^	7.56 (0.72)	2.78 (1.24)	4.93 (0.16)	41.08	<0.001
Arousal^b^	4.90 (0.75)	5.69 (0.59)	1.99 (0.28)	57.12	<0.001
Word frequency^c^^,^^d^	4.70 (0.76)	4.10 (1.51)	4.59 (1.36)	0.33	0.73
Concreteness^d^	3.26 (0.84)	3.57 (1.09)	2.58 (1.17)	1.32	0.30
Number of phonemes^d^	2.20 (0.84)	2.00 (0.71)	2.00 (0.71)	0.12	0.89
Word length^d^	7.20 (2.28)	6.80 (1.30)	6.20 (2.17)	0.33	0.73

a The means of the valence categories were significantly different.

b Positive and negative words were matched, but were higher in arousal than neutral words.

c Natural log transformation.

d For pairwise t-tests between each valence group, all p-values > 0.1.

#### Procedure

In the emotional memory task, the 15 words were presented to each participant in black text on white cards, one card every 2 s, in the same, pseudorandomized order. To protect the emotional words (positive and negative) from primacy and recency effects (Foldi et al., 2003), the word list started and ended with a neutral item. The participants were instructed to read each word out loud and to try to memorize it. Immediately after presenting all words of each trial, the participants were asked to recall as many of the 15 words as possible from memory. There were three learning trials in total. After a delay interval of approximately 20 min (mean = 20.44, *SD* = 4.90), during which participants performed non-verbal tasks, participants were instructed to again recall as many words as possible from memory (delayed recall). Verbal responses were scored as correct or incorrect. The present analyses used the total number of correctly recalled words at *immediate recall* (i.e., total number of correctly recalled words across the three learning trials) and *delayed recall* for each valence category separately. One participant (NC) did not complete the delayed recall task and was therefore excluded from the analyses of delayed recall performance. Thus, for all analyses on delayed recall the *n* was 39.

### MRI measures

#### MRI image acquisition

All participants received a high-resolution, T1-weighted three-dimensional magnetization-prepared rapid acquisition gradient echo (MPRAGE) anatomical imaging scan (*TI* = 1000 ms, *TR* = 2150 ms, *TE* = 3.5 ms, flip angle = 7°, rectangular field of view = 87.5%, acquisition matrix = 256 × 224 mm, voxel size = 1 × 1 × 1 mm). All scans were obtained from the same 3 Tesla MRI scanner (MAGNETOM Allegra, Siemens at the University Hospital Basel). MRI scanning was conducted within 3 months of behavioral testing (mean interval = 2.4 months, *SD* = 2.4 months).

### Analyses

#### Statistical analyses of behavioral data

Group differences on behavioral indices from the emotional memory task were tested with a 3 (group) × 3 (valence) repeated-measures analysis of variance (ANOVA) to compare the effects of valence and diagnostic group at (a) immediate and (b) delayed recall performance. Valence was treated as a within-subjects and diagnosis as a between-groups factor, and age was included in the model as a covariate. The first and last item on the list, which were used to buffer emotional words from primacy and recency effects, were excluded from the behavioral analyses (thus, *n* = three neutral words). The equality of variances at different levels of the repeated factor was tested using Mauchly's test. *Post-hoc* analyses comparing diagnostic groups were conducted using independent samples' *t*-tests. The *t*-tests were Bonferroni-corrected for the number of comparisons conducted {α_corrected_ = 0.05/[3(groups/valence) × 2(time points)] = 0.008}. If Levene's test for equality of variances was violated (*p* = 0.05), a *t*-statistic was conducted which did not assume homogeneity of variances. All statistical analyses of behavioral data were performed with SPSS version 21 (SPSS Inc. IBM company, 2012).

#### ROI analyses

We concentrated *a priori* on the left and right amygdalae as ROIs. Volumes of these structures were acquired with an automatic subcortical segmentation procedure (Fischl et al., 2002) in FreeSurfer v5.0 (Massachusetts General Hospital, Boston, MA; http://surfer.nmr.mgh.harvard.edu; Dale et al., 1999; Fischl et al., 1999). This procedure uses signal intensities and an *a priori* probabilistic atlas as well as information from neighboring voxels to establish an accurate subcortical segmentation (Fischl et al., 2002). This segmentation method has been shown to perform comparably to manual segmentations (Fischl et al., 2002) and has been used in previous studies in AD (Desikan et al., 2006, 2009; Morey et al., 2009). One-way ANOVAs were used to test for differences in the mean volumes of the left and right amygdalae and hippocampi in the three diagnostic groups using age as a covariate. *Post-hoc* comparisons were performed using one-tailed independent samples' *t*-tests.

We tested the eight predictions described in the introduction [i.e., immediate and delayed recall (2) × positive and negative stimuli (2) × left and right amygdalae (2)] using linear regression analyses. Whole brain total gray matter volume and age were included as covariates in the first step to control for the combined effect of head size and overall cortical atrophy and a potential confounding effect of age, respectively. As the hippocampus has a well-established role in episodic memory (Vargha-Khadem et al., 1997; Aggleton and Brown, 1999) and is assumed to interact with the amygdala to give rise to the emotional enhancement effect, we report analogous regression analyses for left and right hippocampal volumes. We did not include the hippocampus and amygdala in the same regression analyses to avoid multicollinearity, as both structures are comparably affected in MCI and in mild AD (Poulin et al., 2011).

The standard alpha-level (α = 0.05) was Bonferroni-corrected for the number of the *a priori* ROIs (*n* = 2; left and right amygdalae) and the number of time points (immediate and delayed recall; *n* = 2). Thus, the adjusted conservative alpha-level was set at 0.01 {α_corrected_ = 0.05/[2(ROIs) × 2(time points)] = 0.01}. For completeness, the analogous linear regression models with the left and right amygdalae and hippocampi as predictors for the performance with all neutral word stimuli are provided in the supplementary results section (see **Supplementary Results**, Table S2). For simplicity, we used the same statistical threshold as in these analyses above. To maximize variability in brain integrity, we collapsed the diagnostic groups in all brain-behavioral analyses. Statistical analyses with ROI data were performed with SPSS version 21 (SPSS Inc. IBM company, 2012).

#### Whole-brain voxel-based morphometry (VBM) analyses

Preprocessing of MPRAGE images was performed with Statistical Parametric Mapping software (SPM8, Wellcome Institute of Cognitive Neurology, www.fil.ion.ucl.ac.uk) in Matlab 2010 (Mathworks Inc., Sherborn, MA; USA). The MRI images were first segmented into gray matter, white matter and cerebrospinal fluid volumes. Gray matter volume misclassifications around the medial temporal lobe were then manually identified by one of the authors (SLK) and the MPRAGEs were segmented again while masking the misclassifications (see Kivisaari et al., 2012 for details). A study-specific template was created with DARTEL (Ashburner, 2007) using the re-segmented gray matter images. The gray matter images were then normalized to the DARTEL template and MNI space, modulated and smoothed with 8 mm FWHM Gaussian kernel. The subsequent whole brain analyses were conducted using the general linear model in SPM8. To determine the relationship between immediate recall and delayed recall memory performance in each valence category and regional brain volume, participants' preprocessed gray matter brain volumes were subjected to two regression analyses using total scores for positive and negative words, at (a) immediate recall and (b) delayed recall. As in the ROI analyses, the groups were collapsed for the VBM analyses. Performance with each valence category (positive and negative words separately) at a single time point was correlated with whole-brain signal intensities while controlling for the performance with the neutral and opposing emotional valence. Age, total gray matter volume (http://www.cs.ucl.ac.uk/staff/G.Ridgway/vbm/get_totals65.m) and performance with neutral words and the opposing valence were included as covariates to account for possible age-related effects, the combined effect of head size and global atrophy, and “baseline” episodic memory performance, respectively. Statistical parametric maps were thresholded at *p* < 0.01 (uncorrected) in all analyses at the voxel-level. Coordinates of peak voxels in clusters surviving a FWE-corrected *p* < 0.05 adjusted for the entire brain are reported in MNI space. Anatomical areas were determined using the AAL atlas in SPM 8 (Tzourio-Mazoyer et al., 2002).

## Results

### Behavioral results

The mean proportions correct for each group and valence category at immediate and delayed recall are reported in Table 3. To compare NC, aMCI, and AD participants' memory performance in each valence category, repeated measures ANOVAs were performed: one for immediate recall and another for the delayed recall. Mauchly's test indicated that the variances at different levels of the repeated factor were spherical at immediate [χ^2^(2) = 4.97, *p* = 0.08] and delayed recall [χ^2^(2) = 4.96, *p* = 0.08]. Thus, uncorrected ANOVA results are reported. At immediate recall, there was a significant main effect of diagnostic group [*F*_(2, 36)_ = 19.57, *p* < 0.001], but no main effect of valence [*F*_(2, 72)_ = 0.24, *p* = 0.79]. The main effect of diagnostic group reflected better performance of NC participants relative to aMCI participants [*t*_(58.56)_ = 3.64, *p* < 0.008] and AD participants [*t*_(85)_ = 8.37, *p* < 0.008], as well as better performance of aMCI participants relative to AD participants [*t*_(76)_ = 3.36, *p* < 0.008]. There was no significant interaction between valence and diagnostic group at immediate recall [*F*_(4, 72)_ = 0.08, *p* = 0.99]. Age significantly predicted immediate recall performance [*F*_(1, 36)_ = 9.20, *p* = 0.004], such that increasing age was associated with poorer performance.

**Table 3:** **Mean proportions correct at immediate and delayed recall for each valence category [mean (standard deviation)] and for each diagnostic group**.

	**Diagnosis**		
	**NC (***n*** = **14**)**	**aMCI (***n*** = **11**)**	**AD (***n*** = **15**)**
**IMMEDIATE RECALL**			
Positive	0.74 (0.10)	0.57 (0.16)	0.40 (0.17)
Negative	0.55 (0.13)	0.39 (0.18)	0.24 (0.16)
Neutral	0.52 (0.19)	0.32 (0.16)	0.16 (0.19)
**DELAYED RECALL**			
Positive	0.63 (0.21)	0.45 (0.37)	0.11 (0.18)
Negative	0.48 (0.28)	0.27 (0.31)	0.08 (0.17)
Neutral	0.51 (0.35)	0.12 (0.22)	0.07 (0.14)

At delayed recall, we found a significant main effect of diagnostic group [*F*_(2, 35)_ = 14.81, *p* < 0.001], as expected, but not of valence [*F*_(2, 70)_ = 0.12, *p* = 0.89]. The group effect reflected better performance of NC participants relative to aMCI participants [*t*_(72)_ = 3.14, *p* < 0.008] and AD participants [*t*_(59.43)_ = 8.10, *p* < 0.008], and better performance of aMCI participants relative to AD participants [*t*_(43.11)_ = 3.20, *p* < 0.008]. The interaction between valence and diagnostic group approached significance [*F*_(4, 70)_ = 2.20, *p* = 0.08] reflecting a weakened emotional enhancement effect in the AD as compared to the other diagnostic groups. There was no significant effect of age at delayed recall [*F*_(1, 35)_ = 1.67, *p* = 0.21].

### ROI results

One-way ANOVAs revealed that the mean volumes of left and right amygdalae and hippocampi differed significantly over the groups: left amygdala [*F*_(2, 36)_ = 10.79, *p* < 0.001; NC > aMCI, NC > AD, both *p*-values < 0.007, aMCI = AD]; right amygdala [*F*_(2, 36)_ = 8.72, *p* = 0.001; NC > aMCI > AD, all *p-values < 0.007*]; left hippocampus [*F*_(2, 36)_ = 18.66, *p* < 0.001; NC > aMCI > AD; all *p*-values < 0.003]; right hippocampus [*F*_(2, 36)_ = 14.69, *p* < 0.001; NC > aMCI > AD, all *p*-values < 0.02]. Regression models were conducted in which the volume of each ROI was used to predict immediate and delayed recall performance with the positive and negative valence groups to determine whether the integrity of the amygdalae and hippocampi were related to the immediate and delayed recall of emotional information (Table 4). These analyses yielded a significant association between left amygdala volume and immediate recall performance of positive and negative words. Immediate recall of positive but not negative words was also significantly predicted by the left hippocampus. Left amygdala volume and bilateral hippocampal integrity significantly predicted the delayed recall of positive words, whereas only right amygdala volume was significantly associated with the delayed recall of negative words.

**Table 4:** **Results of linear regression analyses where gray matter volume in left and right amygdalae and hippocampi predicted immediate and delayed recall performance for positive and negative stimuli**.

	**Positive words**			**Negative words**		
	**β**	***t***	***p***	**β**	***t***	***p***
**IMMEDIATE RECALL**						
Left amygdala	0.47	3.11	**0.004**	0.44	2.87	**0.007**
Right amygdala	0.20	1.05	0.300	0.36	2.02	0.051
Left hippocampus	0.57	3.38	**0.002**	0.43	2.46	0.019
Right hippocampus	0.34	1.67	0.105	0.40	2.07	0.046
**DELAYED RECALL**						
Left amygdala	0.56	3.11	**0.002**	0.45	2.55	0.015
Right amygdala	0.30	1.44	0.16	0.56	2.97	**0.005**
Left hippocampus	0.76	4.39	**<0.001**	0.36	1.79	0.08
Right hippocampus	0.65	3.12	**0.004**	0.53	2.45	0.02

Age and total gray matter volume were entered as covariates. Statistically significant effects at the Bonferroni-corrected level are in bold. Immediate recall degrees of freedom (df) = 39; delayed recall df = 38.

These results suggest that the integrity of the left amygdala is significantly associated with performance with positive and negative words at immediate recall, whereas the integrity of the right amygdala is not associated with immediate recall performance with either valence. At delay we found a lateralized pattern with the delayed recall performance with positive words associated with left and delayed recall performance with negative words with right amygdala volume. Left and right hippocampus volumes were significantly associated only with the delayed recall with positive, but not negative words. To confirm the robustness of these analyses and to ensure the analyses did not suffer e.g., from floor or ceiling effects, we provide scatterplots representing the relationship between the recalled words at immediate and delayed recall and the volume of left and right amygdalae for significant ROI results (see **Supplementary Results**, Figure S1).

### Whole-brain VBM results

#### Immediate recall by valence

**Figure 1:**
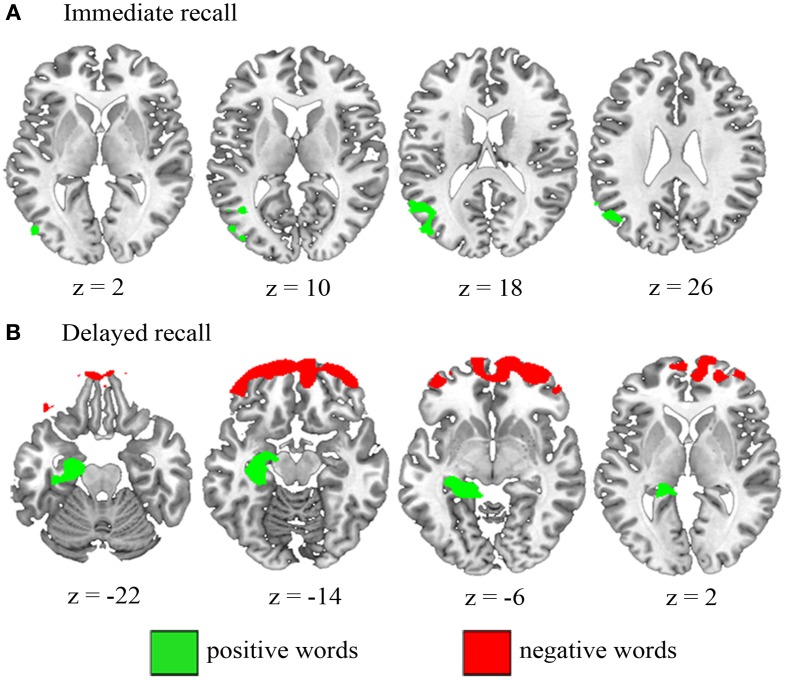
**Neuroanatomical regions where poorer performance at (A) immediate recall and (B) delayed recall was associated with decreased volume by valence.** There were no significant results for negative words at immediate recall. Results are thresholded at *p* < 0.01. MNI coordinates are reported and L = L.

We conducted a whole-brain VBM analysis to examine the entire network of brain regions associated with the immediate recall of words from different valence categories. We tested for brain regions where immediate recall performance with positive and negative items significantly correlated with regional brain volume while controlling for the effects of the opposing valence and neutral words. Immediate recall performance with positive words was associated with one significant cluster centered in the left angular gyrus (−44, −69, 37), which extended into the middle temporal gyrus (Figure 1). There were no significant clusters for the contrast testing for brain regions where reduced volume was associated with poorer immediate recall performance with negative words.

#### Delayed recall by valence

Next, we examined brain regions where delayed recall of positive and negative items was associated with reduced gray matter integrity while controlling for the effects of the remaining valences. Poorer delayed recall of positive words was significantly associated with reduced gray matter volume in one cluster centered in the left hippocampus (−18, −35, −4) extending into the amygdala, perirhinal, entorhinal and parahippocampal cortices (Kivisaari et al., 2013) and the lingual gyrus (Figure 1). Impaired delayed recall of negative words resulted in one significant cluster in the bilateral medial orbitofrontal areas (−31, 62, −15), extending bilaterally into the ventrolateral and -medial prefrontal areas (Figure 1).

The present sample size (*n* = 40 at immediate recall; *n* = 39 at delayed recall) was relatively small compared to conventional VBM studies (cf. Pell et al., 2008), potentially rendering the results susceptible to violations of statistical assumptions, i.e., heteroscedasticity or non-normality of the residuals (Lumley et al., 2002). To test the robustness of these results, we extracted the mean signal intensities for each significant cluster in each VBM model (one cluster centered in the angular gyrus at immediate recall, two clusters at delayed recall centered in the left hippocampus and in the orbitofrontal area). We then replicated each VBM regression analysis in SPSS using the respective mean signal intensities as the dependent variables and examined the residuals of these models. Shapiro-Wilk tests indicated that the standardized residuals were normally distributed [at immediate recall: *D*_(40)_ = 0.97, *p* > 0.3; both clusters at delayed recall: *D*_(39)_ > 0.9, all *p* > 0.4]. Moreover, an examination of the standardized residuals plotted against the standardized predicted values confirmed that these data were homoscedastic. Thus, these analyses support the validity of the VBM multiple regression approach despite the relatively small sample size.

## Discussion

Left amygdala integrity was significantly associated with immediate recall performance with both valences, but a lateralized pattern emerged at delayed recall, such that left and right amygdala were associated with performance with positive and negative words, respectively. The whole-brain analyses testing for regions uniquely associated with the immediate or delayed recall performance with either positive or negative valence revealed additional associations between the left angular gyrus volume and performance with positive words at immediate recall, as well as between the orbitofrontal, bilateral ventrolateral and -medial prefrontal cortex and performance with negative words at delayed recall. Thus, these analyses revealed a network of regions supporting emotional immediate and delayed memory performance and contribute to a better understanding of the neural underpinnings of emotional memory impairments in conditions such as AD that affect the functioning of this system.

At the behavioral level, the groups did not differ in their ability to benefit from stimulus emotionality. This finding is at odds with behavioral studies which found weakened emotional memory effects in AD patients (Ikeda et al., 1998; Moayeri et al., 2000; Boller et al., 2002; Nieuwenhuis-Mark et al., 2009; Nashiro et al., 2011; Perrin et al., 2012; for an overview see Broster et al., 2012; Klein-Koerkamp et al., 2012). It should be noted, however, that the interaction between valence and diagnosis approached significance at delay, reflecting a weakened emotional enhancement of positive vs. neutral items in the AD group as compared to the two other diagnostic groups. The lack of significant group differences in the behavioral analyses may partially be explained by the relatively short time interval between immediate and delayed recall (ca. 20 min), which may have attenuated the differences in emotional enhancement between groups (cf. McGaugh, 2000).

In the ROI regression analyses, the volumes of the left and right amygdalae as well as those of the hippocampi were associated with emotional memory performance at delay. These results are consistent with the well-established finding that the amygdala is critical for the consolidation of emotional material together with its interactions with the hippocampus (Cahill and McGaugh, 1998; McGaugh, 2000; Dolcos et al., 2005). Importantly, also the immediate recall of positive words significantly correlated with left amygdala volume. This result supports the claim that the amygdala enhances emotional memory performance already at immediate recall. The amygdala is thought to serve a facilitatory role at encoding by influencing the direction of attentional focus and increasing the likelihood of perceiving and attending to emotionally salient stimuli, resulting in enhanced memory for these items (Anderson and Phelps, 2001; Phelps et al., 2006; De Martino et al., 2009; Jacobs et al., 2012; but see also Piech et al., 2011; Edmiston et al., 2013). Thus, by facilitating the perception and attention of emotional stimuli, the amygdala promotes hippocampal consolidation of these items (Richardson et al., 2004). The association between the volumes of the left and right amygdalae and the immediate recall of neutral words (see **Supplementary Results**, Table S2) suggests that this region may also facilitate the learning of neutral material, possibly by embedding it in an emotional context (cf. Maratos et al., 2001). Taken together, these findings from the brain-behavioral analyses suggest that the amygdala, together with its interactions with the hippocampus, may underpin the emotional memory impairments in part by facilitating the on-line processing of emotional material (Phelps, 2004; Richardson et al., 2004).

In line with the hemispheric lateralization hypothesis, using a matched set of positive and negative stimuli, we found that the left and right amygdalae were involved in emotional enhancement at delay (cf. Hamann and Mao, 2002). Specifically, the left amygdala significantly predicted delayed recall of positive but not negative items, whereas right amygdala damage was associated with delayed recall of negative but not positive items. This finding is in line with functional imaging studies (Cahill et al., 1996; Canli et al., 1998; Zalla et al., 2000; Tabert et al., 2001; Kensinger and Schacter, 2006) and patient studies (LaBar and Phelps, 1998; Meletti et al., 2003, 2009) and suggests that stimulus valence may indeed modulate the laterality of amygdala involvement. Interestingly, however, we found that performance with both positive and negative words at immediate recall was associated with the left amygdala volume. This result corroborates previous findings suggesting that the left amygdala facilitates the on-line processing of especially verbal emotional stimuli (Anderson and Phelps, 2001; Hamann and Mao, 2002) and may help to explain why the left amygdala is more often associated with the processing of both valences compared to the right amygdala in the previous literature on emotional long-term memory processing (cf. Adolphs et al., 2000; Buchanan et al., 2001; Frank and Tomaz, 2003). That is, while the left amygdala may support the encoding of both positive and negative items as well as the consolidation of positive material, the right amygdala may primarily support the consolidation of negative material. In other words, processing of negative material may be supported by bilateral amygdala as suggested by others (cf. Liberzon et al., 2000; Hamann et al., 2002; Baeken et al., 2009), with the left and right amygdala possibly recruited at different stages of memory processing.

It should be noted that we cannot dissociate the effect of the amygdala shrinkage from that of the hippocampus by linear regression analyses, since both regions are comparably affected in AD (Poulin et al., 2011) and contribute to episodic memory processing. However, a complete dissociation of the two structures in emotional memory processing would not necessarily be expected in a patient study, since the emotional memory enhancement is claimed to be underpinned by the robust interactions between the two regions (Cahill and McGaugh, 1998; Dolcos et al., 2004b; Ritchey et al., 2008). Thus, the present findings support the idea that the amygdala, together with the hippocampal formation, underpins the emotional enhancement effect during episodic memory and processing.

The whole-brain analyses indicated that performance with negative words at delay was significantly associated with the integrity of the bilateral prefrontal cortex, including its ventrolateral and -medial aspects and the orbitofrontal cortex (see also Kumfor et al., 2013). These regions may serve to strengthen the consolidation of such information via controlled processes such as elaboration, focused attention, and associative processing of emotional information (Craik and Lockhart, 1972; Bechara et al., 2000a; Kensinger, 2004). For example, patients with damage to the prefrontal cortex, including the orbitofrontal and ventromedial cortices, have been shown to have deficits in regulating their behavior with respect to expected negative consequences (Bechara et al., 1994, 2000b) and exhibit an attenuated emotional response, such as regret, to unrewarding decisions in a gambling task with a monetary reward (Camille et al., 2004). Thus, the importance of the ventromedial and -lateral and orbitofrontal prefrontal regions for the processing of negative emotions may explain why damage to this region would particularly impair the memory for unpleasant material.

The whole-brain analyses revealed an additional association between the immediate recall of positive words and volume of the left angular gyrus in the parieto-temporal junction. This region has been linked with semantic processing and working memory (Price, 2000; Devereux et al., 2013) and has been shown to be activated during encoding and recognition of emotional (positive and negative) pictures (Smith et al., 2004) and during the viewing of happy facial expressions (Habel et al., 2005). Therefore, this region may support the learning of positive words by means of deeper elaboration and integration of the information into a broader semantic network. The results presented here extend the previous literature by suggesting that this region may also be *necessary* for such processing and for the immediate recall of information that carries positive content.

The involvement of prefrontal structures and the angular gyrus in emotional memory may reflect the importance of these areas in autobiographical processing. Autobiographical memory, in contrast to classical episodic memory tasks, is rich in emotional content (Cabeza and Jacques, 2007; Holland and Kensinger, 2010) and, therefore, may enhance emotional processing by e.g., providing contextual links with high personal relevance. Specifically, the ventral prefrontal region has been implicated in e.g., integrating new stimuli into a specific context of already existing information of past events and binding different aspects of an episode together (Thompson-Schill et al., 1997; Fernández and Tendolkar, 2001), and processing and re-experiencing emotions that were present during learning (Gilboa, 2004; Daselaar et al., 2008). The angular gyrus has been suggested to support autobiographical processing by contributing to the richness and vividness of remembered detailed information of personal events (Berryhill et al., 2007). Thus, damage to these systems associated with autobiographical processing may impair emotional memory by hindering the putative privileged access of emotional information into existing semantic and episodic information.

These brain-behavioral effects reported above cannot be accounted for by overall disease severity as we controlled for the confounding effect of total gray matter volume. Pathology in AD typically affects the amygdala and hippocampus earlier than it affects the frontal cortices (Braak and Braak, 1991). However, a proportion of clinically diagnosed aMCI patients may in fact be in an advanced stage of AD neurofibrillary pathology (Petersen et al., 2006) with tangle deposition in the frontal regions (Braak stages III-V; Braak and Braak, 1991). Therefore, it is possible that subtle emotional memory impairments due to pathology in the frontal cortices are apparent even in clinically early stages of AD such as the patients in this sample. Finally, it should be noted that since we combined the three diagnostic groups for both the ROI regression and whole-brain analyses, the results demonstrate a general brain-behavioral relationship across a broad spectrum of variability, from healthy brain tissue to different stages of disease pathology. We suggest that emotional memory impairments can be accounted for by the degree and location of pathology rather than diagnostic status, such that similar patterns of behavioral impairment would be expected in any neuropathological condition that affects these brain networks.

The present results indicate that emotional memory in mature people and in the context of AD depends on the integrity of a network of brain regions. Specifically, we demonstrate that a distributed network of brain regions comprising the amygdala, the hippocampus, the prefrontal cortex and the angular gyrus, is necessary for the processing of emotional items. Moreover, this study provides evidence for the hemispheric lateralization model. However, the present results, taken together with the previous literature, suggest that the lateralization is relative rather than absolute and may vary at different stages of mnemonic processing. This study accounts for a better understanding of neuroanatomical networks involved in emotional memory deficits in AD and suggests that emotional memory may rely on brain regions implicated in emotional processing as well as frontoparietal regions which may support emotional memory via controlled organizational strategies and autobiographical processing.

## Author contributions

Panagiota Mistridis: data analyses, interpretation, drafting of the manuscript, critical reviews. Kirsten I. Taylor: study design, data acquisition, drafting of the manuscript, interpretation, critical reviews. Johanna M. Kissler: interpretation, critical reviews. Andreas U. Monsch: interpretation, critical reviews. Reto W. Kressig: interpretation, critical reviews. Sasa L. Kivisaari: data analyses, interpretation, drafting of the manuscript, critical reviews. The authors agree to be accountable for all aspects of the work and ensure that the work has been conducted accurately. All authors approved the manuscript before submission.

## Conflict of interest statement

The authors declare that the research was conducted in the absence of any commercial or financial relationships that could be construed as a potential conflict of interest.

## Supplementary material

Table S1 **Summary of neuropsychological test performance (presented as age, gender and education adjusted z-scores) of the participants**.

Table S2 **Results of linear regression analyses where gray matter volume in left and right amygdalae and hippocampi predicted immediate and delayed recall performance for neutral stimuli**.

Figure S1 **Scatterplots representing the relationship between the recalled words at immediate and delayed recall and the volume of left and right amygdalae**.
