# Supplementary material for: Distinct neural systems underlying reduced emotional enhancement for positive and negative stimuli in early Alzheimer's disease
Source: Front Hum Neurosci. 2014 Jan 20;7:939. doi: 10.3389/fnhum.2013.00939 (PMC3895803; doi:10.3389/fnhum.2013.00939)
Supplement: Table S1 — Summary of neuropsychological test performance (presented as age, gender and education adjusted z-scores) of the participants. [file DataSheet1.DOCX]

**Distinct neuroanatomical systems underlying reduced emotional enhancement for positive and negative stimuli in early Alzheimer’s disease**

Panagiota Mistridis^1,2^, Kirsten I. Taylor[^1,3,4^](mailto:1,3,4), Johanna Kissler^5,6^, Andreas U. Monsch^1,2^, Reto W. Kressig^2,3^, Sasa L. Kivisaari^1,4^

^1^ Memory Clinic, University Center for Medicine of Aging Basel, Felix Platter Hospital, Basel, Switzerland

^2^ University of Basel, Basel, Switzerland

^3^ University Center for Medicine of Aging Basel, Felix Platter Hospital, Basel, Switzerland

^4^ Centre for Speech, Language and the Brain, Department of Experimental Psychology, University of Cambridge, Cambridge, UK

^5^ Department of Psychology, University of Bielefeld, Bielefeld, Germany

^6^ Department of Psychology, University of Konstanz, Konstanz, Germany

**Supplementary Material**

**Supplementary Methods**

**Participants**

**Table S1.** Summary of neuropsychological test performance (presented as age, gender and education adjusted z-scores) of the participants.

NC (*n* = 14) aMCI (*n* = 11) AD (*n* = 15) *F* *p*

*N* M (SD) *N* M (SD) *N* M (SD)

**Processing speed**

Trail Making A^a^ 14 0.99 (1.24) 11 -0.22 (1.08) 14 -0.61 (1.54) 5.57 .008

Stroop (color naming)^b^ 13 0.52 (0.70) 11 0.36 (1.01) 15 -0.27 (1.39) 2.05. .144

**Attention**

Digit span forwards^c^ 14 0.31 (1.40) 11 -0.12 (1.05) 15 -0.12 (1.02) 0.62 .544

Corsi block forwards^d^ 14 -0.62 (1.10) 11 -0.59 (0.82) 15 -0.80 (0.74) 0.22 .807

**Verbal Memory**

Learning (CERAD^e^/CVLT^f^) 14 0.35 (0.82) 11 -1.11 (1.00) 15 -2.46 (0.98) 33.71 <.001

Delayed free recall 14 0.46 (0.98) 11 -1.31 (1.25) 15 -2.73 (1.17) 28.86 <.001

Recognition 14 0.18 (1.15) 11 -0.77 (1.00) 15 -2.33 (1.19) 18.30 <.001

**Visual Memory**

ROCF^g^ delayed free recall 10 0.76 (0.83) 6 -1.12 (0.71) 5 -2.14 (1.25) 18.92 <.001

**Language**

BNT^h^ (15-item) 14 0.51 (0.90) 11 -0.52 (1.44) 15 -0.97 (1.41) 5.16 .011

**Executive functions**

Digit span backwards^c^ 14 -0.38 (0.86) 11 -0.35 (1.01) 15 -0.74 (1.06) 0.68 .513

Corsi block backwards^d^ 14 -0.26 (1.01) 11 -0.66 (0.67) 15 -1.29 (0.92) 4.85 .013

Trail Making B^a^ 14 0.86 (1.06) 10 -0.67 (0.94) 15 -0.43 (1.10) 7.70 .002

Trail Making ratio B/A 14 -0.02 (0.90) 10 -0.61 (0.87) 10 -0.68 (0.78) 2.13 .137

Stroop Interference^b^ 14 0.06 (0.69) 11 -0.65 (0.92) 15 -0.94 (0.98) 4.98 .012

Semantic fluency^i^ (animals) 14 0.72 (0.90) 11 -0.65 (0.95) 15 -1.13 (0.81) 16.95 <.001

Phonemic fluency^j^ 14 0.56 (1.18) 11 -0.54 (0.96) 15 -0.57 (1.26) 4.23 .022

(S-words)

^a^ Reitan, 1958.

^b^ Stroop, 1935.

^c^ Wechsler, 1987.

^d^ Wechsler, 1987.

^e^ CERAD = German version of the Consortium to Establish a Registry for Alzheimer’s Disease (Morris et al., 1989).

^f^ CVLT = German version of the California Verbal Learning Test (Delis et al., 1987).

^g^ ROCF = Rey-Osterrieth Complex Figure (Rey, 1944).

^h^ BNT = 15-item version of the Boston Naming Test (Kaplan et al., 1983; Morris et al., 1989).

^i^ Morris et al., 1989.

^j^ Lezak et al., 2004.

**Supplementary Results**

**ROI results**

We used the volumes of bilateral amygdalae and hippocampi to predict immediate and delayed recall performance with neutral stimuli. The results revealed that bilateral amygdalar and hippocampal volumes were associated with immediate recall performance with neutral words, whereas at delayed recall only bilateral hippocampal volumes were significantly associated with performance with neutral words (see Table S2).

**Table S2.** Results of linear regression analyses where gray matter volume in left and right amygdalae and hippocampi predicted immediate and delayed recall performance for neutral stimuli. Age and total gray matter volume were entered as covariates.

**Immediate recall** **Delayed recall**

**β** ***t p-*value β *t p*-value**

**neutral words**

left amygdala 0.50 3.48 **0.001** 0.44 2.67 0.011

right amygdala 0.50 3.00 **0.005** 0.46 2.44 0.020

left hippocampus 0.63 4.11 **<0.001** 0.55 3.03 **0.005**

right hippocampus 0.79 5.10 **<0.001** 0.57 2.84 **0.007**

*Note*. Statistically significant effects at the Bonferroni-corrected level are in bold. Immediate recall degrees of freedom (*df*) = 39; delayed recall *df* = 38.


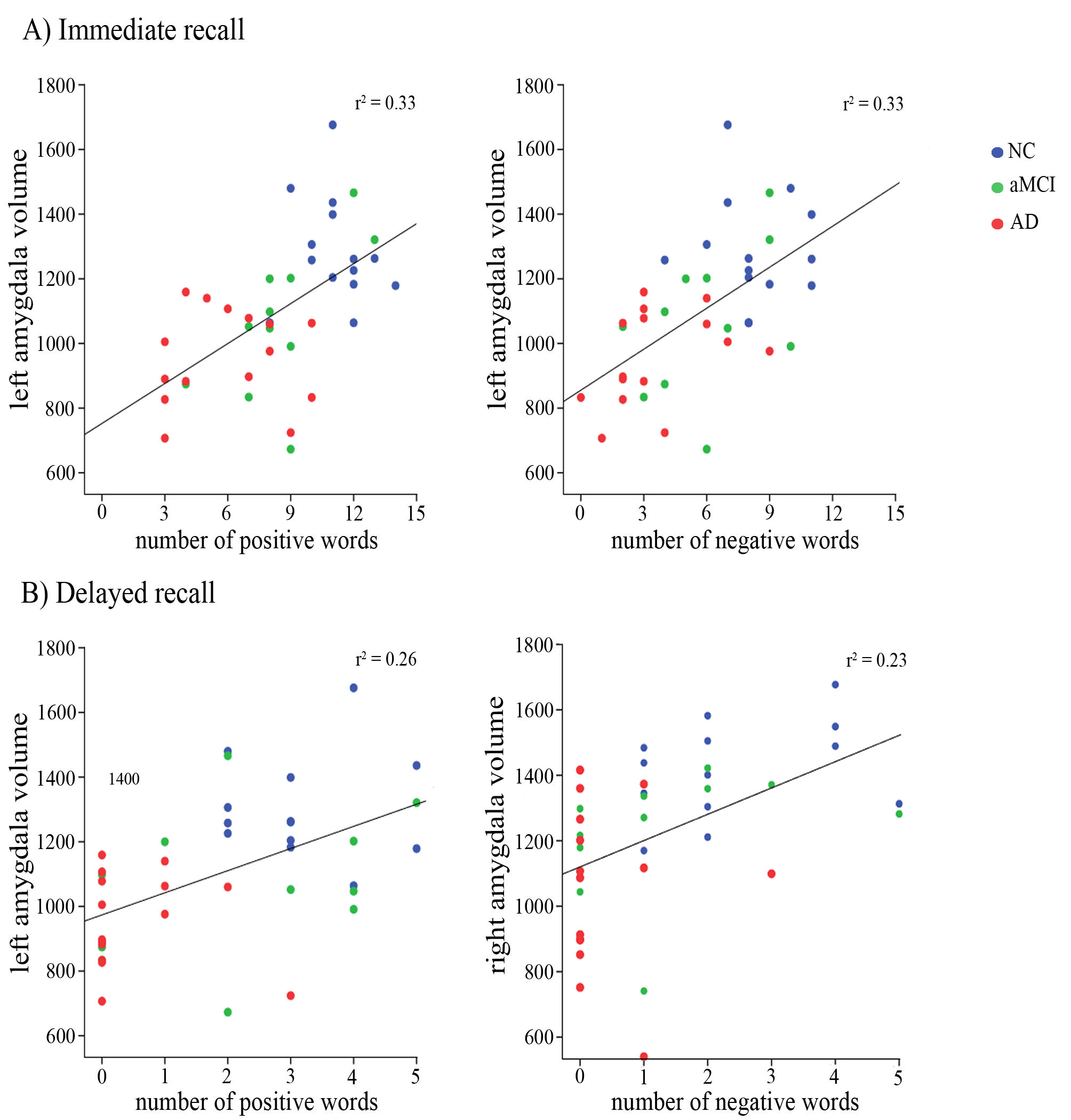


**Figure S1.** Scatterplots representing the relationship between the recalled words at immediate and delayed recall and the volume of left and right amygdalae.

**References**

Delis, D. C., Kramer, J. H., and Ober, B. A. (1987). *California Verbal Learning Test*. San Antonio, TX: Psychological Corporation.

Kaplan, E., Goodglass, H., Weintraub, S., and Segal, O. (1983). *Boston Naming Test*. Philadelphia: Lea & Febiger.

Lezak, M. D., Howieson, D. B., Loring, D. W., Hannay, H. J., and Fischer, J. S. (2004). *Neuropsychological Asessment*. 4th ed. New York, NY: Oxford University Press.

Morris, C. J., Heyman, A., Mohs, R. C., Hughes, J. P., van Belle, G., Fillenbaum, G., Mellits, E. D., and Clark, C. (1989). The consortium to establish a registry for Alzheimer’s disease (CERAD). Part I. Clinical and neuropsychological assessment of Alzheimer’s disease. *Neurology* 39(9), 1159–1165.

Reitan, R. M. (1958). Validity of the trail making test as an indicator of organic brain damage. *Percept. Mot. Skills* 8(3), 271–276. doi:10.2466/pms.1958.8.3.271.

Rey, P. A. (1944). Le test de copie d’une figure complexe; contribution à l’étude de la perception et de la mémoire. [Test of copying a complex figure; contribution to the study of perception and memory.]. *Arch. Psychol.* 30, 206–356.

Stroop, J. R. (1935). Studies of interference in serial verbal reactions. *J. Exp. Psychol.* 18(6), 643–662. doi:10.1037/h0054651.

Wechsler, D. (1987). *Wechsler Memory Scale-Revised*. New York: Psychological Corporation.
